# Monochannel Preference in Autism Spectrum Conditions Revealed by a Non-Visual Variant of Rubber Hand Illusion

**DOI:** 10.1007/s10803-021-05299-9

**Published:** 2021-09-30

**Authors:** Mattia Galigani, Carlotta Fossataro, Patrizia Gindri, Massimiliano Conson, Francesca Garbarini

**Affiliations:** 1 Manibus Lab, Psychology Department, University of Turin, Via Verdi 10, 10124 Turin, Italy; 2 San Camillo Hospital of Turin, Turin, Italy; 3 Neuropsychology Laboratory, Psychology Department, University of Campania Luigi Vanvitelli, Caserta, Italy; 4 Neuroscience Institute of Turin, University of Turin, Turin, Italy

**Keywords:** Autism spectrum conditions, Rubber hand illusion, Multisensory integration, bOdy ownership, Touch

## Abstract

Individuals with autism spectrum conditions (ASC) are less susceptible to multisensory delusions, such as rubber hand illusion (RHI). Here, we investigate whether a monochannel variant of RHI is more effective in inducing an illusory feeling of ownership in ASC. To this aim, we exploit a non-visual variant of the RHI that, excluding vision, leverages only on the somatosensory channel. While the visual-tactile RHI does not alter the perceived hand position in ASC individuals, the tacto-tactile RHI effectively modulates proprioception to a similar extent as that found in typical development individuals. These findings suggest a more effective integration of multiple inputs originating from the same sensory channel in ASC, revealing a monochannel preference in this population.

Autism Spectrum Conditions (ASC) are complex neurodevelopmental conditions characterized by two symptom domains: difficulties in social interaction and communication (social domain) and restricted, stereotyped, and repetitive behaviors (non-social domain) (American Psychological Association, 2013, DSM-5). The growing interest in research on sensory processing in ASC (Cascio et al., 2008, 2016; Hamilton & Pelphrey, 2018; Robertson & Baron-Cohen, 2017) has led to the inclusion of atypical sensory processing among the core diagnostic criteria of the non-social domain (DSM-5). Atypical sensory processing is often observed in unisensory modalities (Baum et al., 2015; DeBoth & Reynolds, 2017; Robertson & Baron-Cohen, 2017); however, individuals with ASC also perform poorly in conditions requiring the integration of multimodal information (Brandwein et al., 2013; Foxe et al., 2015; Russo et al., 2010; Stevenson et al., 2017) (but see Noel et al., 2020; Zaidel et al., 2015 for opposite evidence). Multisensory integration processes in ASC have been often studied with illusions, and, in particular, the visuo-tactile processing has been gauged by exploiting the Rubber Hand Illusion (RHI) (e.g., Cascio et al., 2012). Here, we leveraged on the RHI to explore sensory processing in persons with ASC from a novel perspective, by comparing the classical visuo-tactile version of the RHI, where two sensory modalities are used to induce the illusion, with a “monochannel” variant of the RHI where only somatosensory signals are involved.

In the classical RHI paradigm (from now on, visuo-tactile RHI), participants watch a human-like rubber hand being touched while their own hand, hidden from view, is touched synchronously. After this procedure, subjects often report that they feel the rubber hand as if it was their own hand, as the visual and tactile inputs originate from a common source (Botvinick & Cohen, 1998). This sense of ownership over the fake limb is induced by visuo-tactile spatial–temporal synchrony, which overrides the incongruent proprioceptive information (Tsakiris et al., 2007). Previous research showed less susceptibility to the illusion in ASC than TD individuals (Cascio et al., 2012; Palmer et al., 2013; Paton et al., 2012). Studies on motor coordination and motor learning demonstrated that individuals with ASC depend more heavily on proprioceptive than on visual information when incongruent (Haswell et al., 2009; Izawa et al., 2013; Valori et al., 2020). In line with this, the reduced susceptibility to RHI in ASC individuals may reflect a stronger tendency to focus on proprioceptive signals in the presence of competing signals from other modalities, thus making the proprioceptive system less vulnerable to bias originating from the visuo-tactile conflict. Likewise, Paton and co-authors’ study (Paton et al., 2012) revealed that adults with ASC displayed not only reduced embodiment of the rubber hand, but also a greater precision in estimating the position of their hidden hand as compared to TD individuals, indicating a bias towards proprioceptive processing.

Besides the over-reliance on proprioception hypothesis, we may assume that the less susceptibility to the RHI in ASC may rely on the experimental procedure used to induce the illusion that exploits two sensory channels (i.e., visual and tactile). In this way, the less effectiveness in integrating these two sources of stimuli in ASC individuals may prevent the emergence of the illusion. Accordingly, perception processing in persons with ASC has been proposed to be ‘monochannel’, suggesting that attention directed towards one sensory modality may impair the ability to perceive and attend to another (Bahrick & Todd, 2012; Hill et al., 2012). Consistent with this hypothesis, persons with ASC may have impaired sensory processing in noisy environments (i.e., those with a high degree of sensory information), so that ASC individuals focus on information from one sensory channel to the detriment of other channels to reduce feelings of sensory overloads (Mongillo et al., 2008; Park et al., 2017). Therefore, we can hypothesize that a “monochannel” variant of the RHI would be more effective in inducing an illusory feeling of ownership over the fake hand in ASC, thus allowing us to demonstrate an efficient integration of multiple sensory sources when they involve the same channel. To this aim, we exploited a non-visual variant of the RHI (Ehrsson et al., 2005; Lopez et al., 2012; Nava et al., 2017) that, excluding vision, leverages only on the somatosensory channel to induce the illusion. During this version of the RHI (from now on, tacto-tactile RHI), participants are blindfolded, and the experimenter strokes the participants’ left hand while holding the participants’ right hand to stroke the rubber hand. In this way, tactile inputs coming synchronously from both participants’ hands should be integrated into a unique sensory percept, affecting proprioception precision and making participants to feel as if the tactile sensation is coming from the fake hand. We recruited two groups of adolescents with a comparable ASC severity. The first group underwent the classical visuo-tactile RHI, whereas the second group underwent the tacto-tactile RHI. Moreover, we enrolled two groups of TD adolescents as control samples. According to the ‘monochannel’ hypothesis, we expect to confirm the reduced susceptibility to visuo-tactile RHI, but to observe the emergence of the illusion after the tacto-tactile RHI. Importantly, if verified, our hypothesis will reveal that the monochannel preference, together with the other mechanisms proposed in previous studies (Cascio et al., 2012; Greenfield et al., 2015; Paton et al., 2012), plays a relevant role in explaining sensory impairments in ASC. Alternative results, showing the same extent of susceptibility to tacto-tactile and visuo-tactile RHI in ASC, would instead challenge our hypothesis. Thereby, these findings would highlight that the reduced susceptibility to the illusion is mainly driven by other factors, such as the over-reliance on proprioception as indicated by previous research (Greenfield et al., 2015; Paton et al., 2012).

## Methods

### Participants

A total of 102 right-handed participants (age range: 9–16; 51 high-functioning ASC and 51 TD) took part in the study; children with ASC were recruited at the “San Camillo” Hospital (Turin, Italy). The subjects’ handedness was evaluated with the Flinders Handedness survey (FLANDERS) (Nicholls et al., 2013).

Twenty-five children with ASC and twenty-five TD controls underwent the visuo-tactile RHI; A different sample of twenty-six children with ASC and twenty-six TD controls were enrolled in the tacto-tactile RHI (see Table 1 for sample characteristics). Despite acknowledging that a within subjects’ study would have been more elegant, we opted for a between design due to logistic issues, and we enrolled different subjects for each RHI version. Since we recruited ASC individuals during after school activity, we decided to administer only one session of the experiment per subject in order to not weigh on adolescents and families asking them relevant extra time.

**Table 1:** Sample characteristics

RHI	Group	N	Age	IQ	% Female
**VT**	ASC	25	13.12 ± 2.44	114.50 ± 3.84	24
	TD	25	12.36 ± 1.68	114.94 ± 3.46	40
**TT**	ASC	26	13 ± 2.47	114.50 ± 3.41	23
	TD	26	12.13 ± 1.65	113.37 ± 2.43	23

*VT* visuo-tactile RHI, *TT* tacto-tactile RHI, *ASC* autism spectrum condition, *TD* typically developing. Age and IQ values reflect the mean and standard deviation

Diagnosis of ASC was reached after a multidisciplinary assessment by a neuropsychiatrist and a clinical psychologist trained in evaluating individuals with neurobehavioral disorders according to DSM-V criteria. Clinical diagnosis was validated by means of the Autism Diagnostic Interview-Revised (ADI-R; C. Lord et al., 1994) and the Autism Diagnostic Observation Schedule (ADOS; Catherine Lord et al., 2000) administered by trained and certified examiners.

Individuals with a history of epilepsy, neurological abnormalities, genetic syndromes, general learning disability, significant head injury, or psychosis were excluded from the study. Cognitive level of all participants was measured by the Raven’s Progressive Matrices and estimated IQ were obtained (Coloured Raven’s Progressive Matrices for 9-to-10-year-old participants, Raven, 1954); Standard Raven’s Progressive Matrices for 11-to-16-year-old participants, (Maria et al., 2009). Independent samples t-tests showed that age and estimated IQ did not differ between ASC and TD individuals that underwent visuo-tactile RHI, between ASC and TD individuals that underwent tacto-tactile RHI, and between the two groups of ASC and the two group of TD [age: visuo-tactile RHI, ASC vs TD: t(1,48) = 1.283, p = 0.205; tacto-tactile RHI, ASC vs TD: t(1,50) = 1.657, p = 0.104; visuo-tactile ASC vs tacto-tactile ASC: t(1,49) = 0.175, p = 0.862; visuo-tactile TD vs tacto-tactile TD: t(1,49) = 0.692, p = 0.492] [IQ: visuo-tactile RHI, ASC vs TD: t(1,48) = 0.463, p = 0.645; tacto-tactile RHI, ASC vs TD: t(1,50) = 1.364, p = 0.178; visuo-tactile ASC vs tacto-tactile ASC: t(1,49) = 0.019, p = 0.984; visual-tactile TD vs tacto-tactile TD: t(1,49) = 1.883, p = 0.066].

All the participants completed the experimental tasks that were approved by the local ethical committee (Ethical Committee of the ASLTO1 of Turin; N:46485/13) and were conducted according to the Helsinki Declaration. All the participants’ parents (as they were minors) provided written informed consent.

### Experimental Procedure

**Fig. 1:**
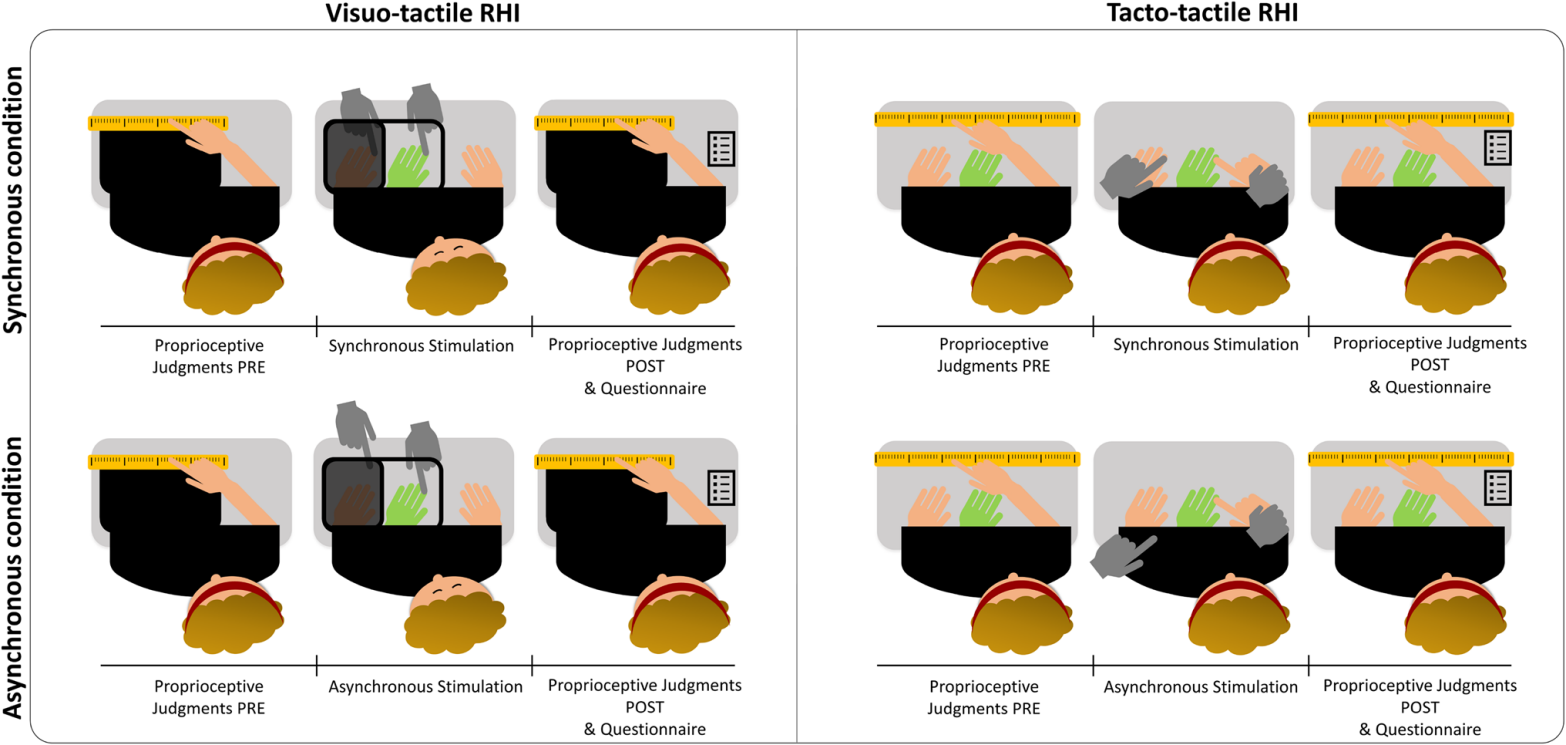
Experimental task & procedure. Both RHI procedures began with a pre-test baseline block of 6 proprioceptive judgments, during which participants had to indicate with their right hand where they felt their left index finger by pointing on a ruler in front of them. The pre-test proprioceptive judgments were followed by 180 s of stroking, which could be either Synchronous (top panels) or Asynchronous (bottom panels), according to the condition. Then, after the stroking period, a post-test block of 6 proprioceptive judgments and the subjective questionnaires were collected. Visuo-tactile RHI (Left Panel), the experimenter’s hands (in grey) stimulated the unseen participants’ left hand synchronously/asynchronously with the rubber hand (the green one). Tacto-tactile RHI (Right Panel), participants were blindfolded, the left experimenter’s hand (in grey) stroked the participants’ left hand, while the right experimenter’s hand was holding the participants’ right hand to stroke the rubber hand (in green), synchronously/asynchronously according to the condition (colour figure online)

In the visuo-tactile RHI (Fig. 1—left panel), we employed a wooden box (60 × 40 × 20 cm) divided into two equal parts (30 × 30 cm) by a panel. One of the two parts was open to the view to allow viewing the rubber hand, while the other half was always covered to take out of sight the real subject’s hand. Two square holes (12 × 12 cm) on both horizontal sides of the box allowed placing the participant’s arm and the rubber hand (Fossataro et al., 2018a, 2018b). A black towel covered the subject’s shoulders and the proximal end of both the subject’s real hand and the rubber hand so that the rubber hand was perceived as an extension of the participant’s own arm. The box was placed in front of the subject’s chest (about 15 cm far) and set to have the rubber hand, placed in the half of the box open to the view, aligned with the participant’s shoulder. Participant placed her left arm within the part of the box hidden to the view, with the palm facing down and the fingers stretched out. In the other half of the box, open to the view, a left rubber hand was placed (at a distance of approximately 25 cm from the own hand), exactly where the participant’s hand has to be. The hand stroking (Synchronous or Asynchronous) was delivered for 180 s by the experimenter’s hand on the index finger from the knuckle to the fingertip, at an approximate frequency of 1 Hz. An in-ear metronome controlled the stimulation frequency. Asynchronous stroking of the own hand and the rubber hand was utilized as a control condition, in which strokes were delivered spatially and temporally out of phase between the two hands. The order of synchronous and asynchronous stroking was counterbalanced between participants.

In the tacto-tactile RHI (Fig. 1—right panel), the participants were blindfolded, and their left hand was placed at a pre-defined position on the table, externally misaligned with respect to the participants’ shoulder, and a left rubber hand was placed 15 cm (distance between the two index fingers) to the right of the participant’s left hand. The experimenter held the participant’s right hand and used the participant’s right index finger to stroke the rubber hand on its index finger. The experimenter stroked with his index finger the index finger (i.e., from the knuckle to the fingertip) of the participant’s left hand to create the corresponding tactile input. We manipulated the synchrony between the touch applied to the participant’s left hand and the rubber hand, as previously done for the visuo–tactile RHI. The touch was applied either synchronously or asynchronously for 180 s. As for visuo-tactile RHI, synchronous and asynchronous stroking were counterbalanced between participants.

To evaluate the susceptibility to RHI, we collected two measures (i.e., proprioceptive drift and embodiment questionnaire). As objective measure, the proprioceptive drift was calculated as the difference between the perceived position of the index finger collected before (i.e., 6 trials of pre-test baseline proprioceptive judgments) and after (i.e., 6 trials of post-test proprioceptive judgments) the RHI stroking period. During the proprioceptive judgments in the two versions of RHI, both the participant’s real hand and the rubber hand were out of view and the participants were blindfolded. In the visuo-tactile RHI, a ruler was positioned over the box, at the same gaze depth as the rubber hand, and participants were asked to judge the location of their left index finger, by pointing with their right index finger on the ruler to the felt location of their left index finger. In the tacto-tactile RHI, participants were asked to indicate the perceived position of their left index finger by pointing with their right index finger on the ruler placed over a rectangular support, located above the subject’s real hand and the rubber hand (see Fig. 1). Before starting, participants were familiarized with the setting and instructed to all procedures and rating scales. As subjective measure, a questionnaire investigating the feeling of ownership over the fake hand was administered. For the visuo-tactile RHI, we selected three statements from the original study (Botvinick & Cohen, 1998), as it has been done in previous works (della Gatta et al., 2016; Fossataro et al., 2018b; Kanayama et al., 2017). Analogously, from Lopez et al. (2012) we chose the three complementary items for the tacto-tactile RHI (Table 2).

**Table 2:** Each questionnaire consisted of three selected statements adapted from two previous studies (Fossataro et al., 2018b for visuo-tactile RHI; Lopez et al., 2012 for tacto-tactile RHI)

Embodiment questionnaire	
Visuo-tactile RHI	Tacto-tactile RHI
It seemed as if the touch I felt was caused by the finger touching the rubber hand	I felt as if I was touching my left hand with my right index finger
It seemed as if I were sensing the touch of the finger where I saw the rubber hand touched	It seemed as if my hand was positioned where the rubber hand was
I felt as if the rubber hand was my hand	I felt as if the rubber hand was my hand

Participants were asked to evaluate the vividness of their experience of ownership over the rubber hand using a 7-point Likert scale, by rating their agreement/disagreement with each item (− 3 = strong disagreement; + 3 = strong agreement; 0 = neither agreement nor disagreement)

In both questionnaires, participants were asked to evaluate the vividness of their experience of ownership over the stimulated hand using a printed 7- points Likert scale, by rating their agreement/disagreement with each item (− 3 = strong disagreement; + 3 = strong agreement; 0 = neither agreement nor disagreement) presented in random order to avoid learning effects.

Importantly, before starting the experiment, we carefully verified that participants fully understood the procedure and the task.

### Statistical Analyses

In both visuo-tactile and tacto-tactile RHI, the embodiment questionnaire was analyzed as scores calculated as the mean value of the three items (all data have been made available at the following public repository: https://data.mendeley.com/datasets/s82gtf44w6/1). The Proprioceptive Drift was calculated as the difference between the post-test and the pre-test proprioceptive judgments (i.e., the indicated location of the participant index finger before and after the stroking period). For each subject, the six differences obtained from the six pointings were averaged. Embodiment questionnaire scores and Proprioceptive drift values were entered separately in two 2*2*2 repeated measures ANOVAs with Stimulation (two levels: synchronous; asynchronous) as within-subject factor, and RHI (two levels: visuo-tactile group; tacto-tactile group) and Group (two levels: ASC group; TD group) as between-subject factors. Post hoc comparisons were performed by means of Duncan’s test. We tested for homogeneity of variance for between-Group factor (embodiment questionnaire: p = 0.123, drift: p = 0.347).

Furthermore, to rule out that possible group difference is due to a difference between pre-test scores, we performed two 2*2*2 repeated measures ANOVAs (one for visuo-tactile RHI, and one for the tacto-tactile RHI) with Stimulation (two levels: synchronous; asynchronous) and Time (two levels; pre; post) as within-subject factors, and Group (two levels: ASC group; TD group) as between-subject factor.

## Results

**Fig. 2:**
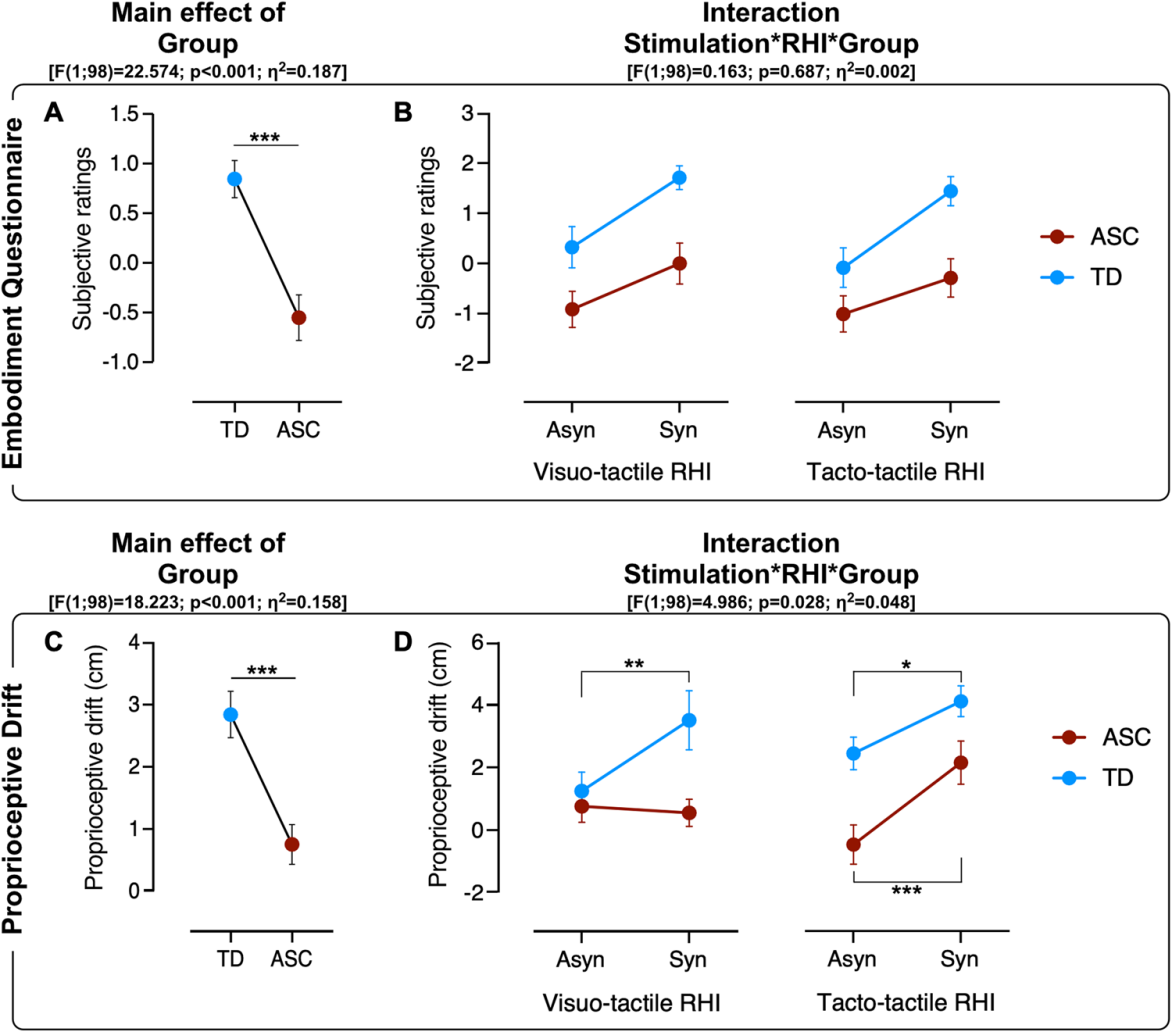
Experimental results. The top panel represents the mean subjective ratings after the visuo-tactile (top-left panel), and after the tacto-tactile (top-right panel) RHI. The bottom panel represents the mean proprioceptive drift after the visuo-tactile (bottom-left panel), and after the tacto-tactile (bottom-right panel) RHI. Panels A and C represent the Main effect of Group, whereas the panels B and D represent the Stimulation*RHI*Group interaction. Significant levels: *p < 0.05; **p < 0.005; ***p < 0.0005. Bars indicate standard errors of the mean (*SEM*)

The 2*2*2 ANOVA performed on Embodiment Questionnaire scores revealed a main effect of Stimulation [F(1;98) = 30.164; p < 0.001; η^2^_p_ = 0.235], with greater scores in synchronous (mean ± SEM:0.71 ± 0.189) than asynchronous (mean ± SEM: -0.42 ± 0.20) stimulation, and a main effect of Group [F(1;98) = 22.574; p < 0.001; η^2^_p_ = 0.187], with a higher agreement showed by TD (mean ± SEM:0.85 ± 0.26) as compared to ASC (mean ± SEM:-0.55 ± 0.27) individuals. See Fig. 2 and Table 3. Conversely, the main effect of RHI was not significant [F(1;98) = 0.733; p = 0.394], as well the interactions Stimulation*Group [F(1;98) = 2.398; p = 0.125], Stimulation*RHI [F(1;98) = 0.005; p = 0.946], RHI*Group [F(1;98) = 0.064; p = 0.800], and Stimulation*RHI*Group [F(1;98) = 0.163; p = 0.687]. The 2*2*2 ANOVA run on Proprioceptive Drift showed a main effect of Stimulation [F(1;98) = 15.048; p < 0.001; η^2^ = 0.148], with a greater shift towards the rubber hand after synchronous (mean ± SEM:2.58 ± 0.36 cm) than after asynchronous stimulation (mean ± SEM:0.99 ± 0.30 cm). Moreover, we found a main effect of Group [F(1;98) = 18.223; p < 0.001; η^2^_p_ = 0.158], with a higher Proprioceptive Drift displayed by TD (mean ± SEM: 2.83 ± 0.48 cm) as compared to ASC (mean ± SEM: 0.75 ± 0.42 cm) individuals. Conversely, the main effect of RHI was not significant [F(1;98) = 1.239; p = 0.268], as well the interactions Stimulation*Group ([F(1;98) = 0.985; p = 0.323], Stimulation*RHI [F(1;98) = 2.112; p = 0.149], and RHI*Group [F(1;98) = 0.584; p = 0.461].

**Table 3:** The table (top panel: embodiment questionnaire; bottom panel: proprioceptive drift) includes the mean values ± SEM of each factor

Embodiment questionnaire		
Stimulation	Synchronous	0.71 ± 1.89
	Asynchronous	− 0.42 ± 0.20
RHI	Visuo-tactile	0.28 ± 0.20
	Tacto-tactile	0.02 ± 0.20
Group	TD	0.84 ± 0.18
	ASC	− 0.55 ± 0.30

Proprioceptive drift		
Stimulation	Synchronous	2.58 ± 0.36
	Asynchronous	0.99 ± 0.30
RHI	Visuo-tactile	1.51 ± 0.34
	Tacto-tactile	2.05 ± 0.33
Group	TD	2.82 ± 0.34
	ASC	0.74 ± 0.30

Crucially, a significant Stimulation*RHI*Group interaction was found [F(1;98) = 4.986; p = 0.028; η^2^_p_ = 0.048], showing that while in TD individuals the effectiveness of the illusion was comparable in visuo-tactile and tacto-tactile group, in ASC individuals the illusion occurred only in tacto-tactile group. Post-hoc analyses showed that in TD individuals the Proprioceptive Drift was significantly greater in synchronous than asynchronous stimulation in both tacto-tactile (p = 0.042) and visuo-tactile (p = 0.007) group. Conversely, in ASC individuals the Proprioceptive Drift was significantly greater in synchronous than asynchronous only in the tacto-tactile group (p = 0.002), but not in the visuo-tactile group (p = 0.784). See Fig. 2.

Then, we performed two ANOVAs to ensure that the group differences in proprioceptive drift scores was not merely driven by differences in the pre-test. As expected, the 2*2*2 ANOVA performed on scores of visuo-tactile RHI revealed a significant Stimulation*Time*Group interaction [F(1;48) = 4.512; p = 0.039]. Post-hoc comparisons confirm the results of the main analysis by revealing significant differences between pre- and post- test scores after synchronous stimulation in the TD (p < 0.001), but not the ASC group (p = 0.411). Crucially, pre-test scores did not differ between groups (pre asynchronous: p = 0.556; pre synchronous: p = 0.846). Moreover, the 2*2*2 ANOVA performed on scores of tacto-tactile RHI did not show a significant Stimulation*Time*Group interaction [F(1;50) = 0.985; p = 0.326], but revealed a significant Stimulation*Time interaction [F(1;50) = 19.941; p < 0.001], thus confirming the main results with a significant difference between pre- and post-test scores after the synchronous (p < 0.001), but not the asynchronous stimulation (p = 0.22), irrespective of group.

## Discussion

Several studies on ASC describe the atypical multisensory processing as a critical component of this complex neurodevelopmental disorder (for a review, see Baum et al., 2015). In the present study, we compared two different versions of the RHI paradigm to investigate whether, in ASC, the integration of multiple sensory sources is more effective when they involve the same channel. In the classical visuo-tactile version of the RHI, participants feel the tactile stimulation as originating from their own hand, but simultaneously see the dummy hand being stroked. This conflict between tactile and visual inputs is usually resolved with the visual capture of touch that leads to an altered proprioception precision and illusory ownership over the rubber hand (Botvinick & Cohen, 1998; Bucchioni et al., 2016; Burin et al., 2017; Fossataro et al., 2018b; Limanowski et al., 2014). In the monochannel variant of RHI, namely the tacto-tactile RHI, the illusory ownership over the rubber hand can be triggered in the absence of visual input by applying tactile stimuli with temporal–spatial congruency (Ehrsson et al., 2005; Lopez et al., 2012). The current experiment demonstrates that, in ASC individuals, exploiting only one sensory modality (i.e., touch) to induce the RHI results in a greater susceptibility to the illusion as compared to the classical bimodal visuo-tactile RHI, which is known to be less effective in inducing ownership over a fake hand in this neurodevelopmental disorder (Cascio et al., 2012; Palmer et al., 2013; Paton et al., 2012).

As far as concerns the subjective feeling of ownership, the embodiment questionnaire analysis revealed significant differences between ASC and TD individuals, irrespective of the RHI procedures and type of RHI, showing that the overall average responses were greater in TD than in ASC sample. Furthermore, we can observe the same pattern of results in the two groups, with greater agreement scores after the synchronous than the asynchronous stimulation. However, it is noteworthy to point out that ratings in the ASC group never displayed a positive agreement. Although ASC individuals proved to be perfectly able to understand task instructions, we could speculate that lack of positive agreement, a finding fully consistent with previous literature (Cascio et al., 2012), was partially related to the over-literal interpretation of language usually characterizing high-functioning autism (Kalandadze et al., 2018; Martin & McDonald, 2003; Moseley & Pulvermüller, 2018). For instance, when asked “I felt as if the rubber hand was my hand”, an over-literal interpretation could have resulted in thinking that a rubber hand can never be their real hand, thus providing disagreement or neutral answers.

As far as concerns the proprioceptive drift, we found a main effect of Group, which indicates an overall greater shift towards the rubber hand in the TD as compared to the ASC group. This finding is in line with previous studies that pointed out an overreliance on proprioception in ASC individuals, which is reflected in a stronger tendency to focus on proprioceptive signals in the presence of competing signals from other modalities (Cascio et al., 2012; Greenfield et al., 2015; Paton et al., 2012; Ropar et al., 2018).

More interestingly, and in line with our hypothesis, the visuo-tactile procedure did not shift the perceived hand position toward the fake hand in ASC individuals, as revealed by the absence of significant differences between synchronous and asynchronous stimulation. On the contrary, the tacto-tactile procedure effectively modulated the proprioception in this population to a similar extent as that found in TD individuals. Indeed, in both groups the proprioceptive drift was significantly greater after synchronous than asynchronous stimulation, thus revealing the emergence of the illusion. In our ASC sample, we can assume that exploiting only the tactile modality enhanced the sensory integration mechanism underpinning the illusion, as compared to the visuo-tactile procedure, wherein autistics participants failed to integrate visual and tactile information regardless the synchronicity of the two (visual and tactile) inputs. Hence, the spatial–temporal congruency between tactile signals from both hands evoked the proprioceptive drift, indicating that the two sources of tactile information originating from two different locations of participants’ body (i.e., right and left hands) was merged and integrated into a single percept. Our significant results are in line with the tenet that ASC perception is locally and selectively oriented, thus yielding a poor performance when it is necessary a global and integrative approach (Bahrick & Todd, 2012). Therefore, persons with autism may have sensory processing difficulties in interacting with sensory-rich environments, and they may focus on information from one sensory modality at the expense of other modalities to reduce the sensory overload (Bertone et al., 2005; Booth & Happé, 2018; Mongillo et al., 2008, but see also Baum et al., 2015). Accordingly, Collignon and coauthors (Collignon et al., 2013) revealed that individuals with ASC have superior unimodal performance but an impaired bimodal performance in a visual search task compared to TD controls. In that study, participants were asked to detect a target line segment that changed color among distractors, and the color change could be accompanied or not by a tone. Crucially, ASC individuals did not benefit from the presence of a typically facilitatory auditory cue but, on the contrary, performed better in the absence of the tone. Authors interpreted these findings as reflecting the inability of ASC individuals to use multiple available sensory sources, thus preferring autonomous sensory processing. In a similar vein, ASC do not benefit as much as TD individuals from the addition of visual information while performing a speech-in-noise task (Foxe et al., 2015) and an audio-visual lip-reading task (Smith & Bennetto, 2007). Indeed, in TD but not in ASC individuals, visualizing a speaker’s articulations considerably improves speech intelligibility under noisy listening conditions.

To conclude, we can assume that the presentation of two stimuli from the same sensory channel allowed ASC individuals to better focus on sensory stimulation, thus inducing a more efficient integration processing highlighted by the emergence of the illusion. In particular, the unisensory stimulation could have partially overridden the over-reliance on proprioception, rendering the proprioceptive percept more susceptible to the bias resulting from the incongruency between tactile inputs.
